# Opioids and Chronic Pain: Impact of the NIH Pathways to Prevention Evidence-Based Workshop Program

**DOI:** 10.1007/s11121-023-01563-9

**Published:** 2023-07-15

**Authors:** Carrie N. Klabunde, Liberty Walton, Keisha L. Shropshire, Luis F. Ganoza, Jen Hession, Kat Schwartz, Elizabeth Vogt, David A. Thomas, Wendy B. Smith, Melissa C. Green Parker, Charlene L. Liggins

**Affiliations:** 1 Office of Disease Prevention, Office of the Director, National Institutes of Health, 6705 Rockledge Drive, Room 733 MSC 7990, Bethesda, MD 20892 USA; 2 Office of Research On Women’s Health, Office of the Director, National Institutes of Health, Bethesda, MD USA; 3 Office of Behavioral and Social Sciences Research, Office of the Director, National Institutes of Health, Bethesda, MD USA

**Keywords:** National Institutes of Health, Pathways to prevention, Opioids, Chronic pain, Impact assessment

## Abstract

The National Institutes of Health (NIH) Office of Disease Prevention (ODP) sponsors Pathways to Prevention (P2P), an evidence-based scientific workshop program that helps advance prevention research. Each P2P workshop is presided over by an independent expert panel and informed by a systematic evidence review, scientific presentations, and public input. Post-workshop activities include collaborating with federal agency partners to develop an action plan for addressing key research gaps. Primary outcomes of P2P workshops include developing a research agenda and creating or enhancing initiatives to implement the agenda. In 2014, ODP partnered with the NIH Pain Consortium and two NIH institutes to convene “The Role of Opioids in the Treatment of Chronic Pain.” This workshop assessed the state-of-the-science on the long-term effectiveness, safety, and harms of opioid use for managing chronic pain. In 2021, ODP initiated an assessment of the outcomes and impact of the Opioids P2P workshop. We applied an evaluation framework and a mixed methods approach encompassing web analytics, bibliometric assessment, grant portfolio analysis, policy assessment, and key informant interviews. Our data showed that the workshop attracted a broad audience, and its published reports had high impact. The workshop also helped inform over 100 new research projects through grants funded by three federal agencies, as well as national legislation and practice guidelines from influential organizations. In sum, the Opioids P2P workshop and follow-up activities have identified gaps in scientific knowledge, informed clinical practice, and catalyzed change on a national level for addressing the prescription opioid crisis.

**Supplementary Information:**

The online version contains supplementary material available at 10.1007/s11121-023-01563-9.

## Introduction

The National Institutes of Health (NIH) Office of Disease Prevention (ODP) sponsors Pathways to Prevention (P2P), an evidence-based scientific workshop program that aids in advancing ODP’s prevention research mission. Located in the NIH Office of the Director, ODP works across NIH Institutes and Centers and with other partners to increase the scope, quality, and impact of prevention research (National Institutes of Health Office of Disease Prevention, 2022a). Through P2P, ODP develops workshops designed to synthesize and interpret evidence and identify research gaps in important areas of public health and prevention. Target audiences for P2P workshops include federal agency staff, researchers, providers, and community members. Each workshop entails a systematic evidence review conducted by an Agency for Healthcare Research and Quality (AHRQ)-supported evidence-based practice center, presentations by expert speakers, public input, and deliberations by an unbiased, independent panel of distinguished scholars who consider the evidence and perspectives presented and write a report with recommendations for advancing the field. After each workshop, ODP convenes a meeting of federal agency representatives to discuss opportunities for collaboration and an action plan. Primary workshop outcomes include developing a research agenda and creating or enhancing initiatives for its implementation (National Institutes of Health Office of Disease Prevention, 2022b).

A critical public health topic addressed by the P2P program was the use of opioids to treat chronic pain. An estimated 25 million Americans experience chronic pain that limits their activities or reduces their quality of life, and 5–8 million use opioids for long-term chronic pain management (Reuben et al., 2015). Opioid use increased dramatically since the 1990s and contributed to substantial increases in addiction and overdoses (Cerdá et al., 2021). Identifying optimal ways of managing chronic pain, including appropriate use of opioids, is a timely and pressing issue. ODP—in collaboration with the NIH Pain Consortium and two NIH institutes—convened the P2P workshop, “The Role of Opioids in the Treatment of Chronic Pain,” on September 29–30, 2014. The workshop assessed the following: 1) long-term effectiveness of opioids, 2) safety and harms of opioid use in patients with chronic pain, 3) effects of different opioid management strategies, and 4) effectiveness of risk mitigation strategies for opioid treatment. A seven-member independent panel presided over the workshop, which was informed by over 20 expert speakers, a systematic evidence review conducted by the Pacific Northwest Evidence-based Practice Center, and a large public audience of in-person and online attendees. Three primary reports from the workshop were published: the independent panel’s report (Reuben et al., 2015) and brief and full evidence reports summarizing the systematic evidence review (Chou et al., 2014, 2015). A federal partners’ meeting report was also published (National Institutes of Health Office of Disease Prevention, 2016).

ODP systematically monitors NIH investments in prevention research and the progress and results of that research (National Institutes of Health Office of Disease Prevention, 2021). As the planning, implementation, and follow-up processes for each P2P workshop require substantial resources—including time, staff, and funding to support the systematic evidence review, meetings, and travel—P2P workshops represent investments meriting assessment of impact. Consistent with these priorities, ODP assessed the outcomes and impact of the Opioids P2P workshop in 2021. The project was guided by four evaluation questions: to what extent did the workshop (Q1) reach its target audience, (Q2) impact funding for research on the role of opioids in chronic pain treatment, (Q3) advance the field of research in opioid use for treating chronic pain, and (Q4) stimulate collaborations. This report describes the methods and main findings of the assessment.

## Methods

### Overview

**Fig. 1:**
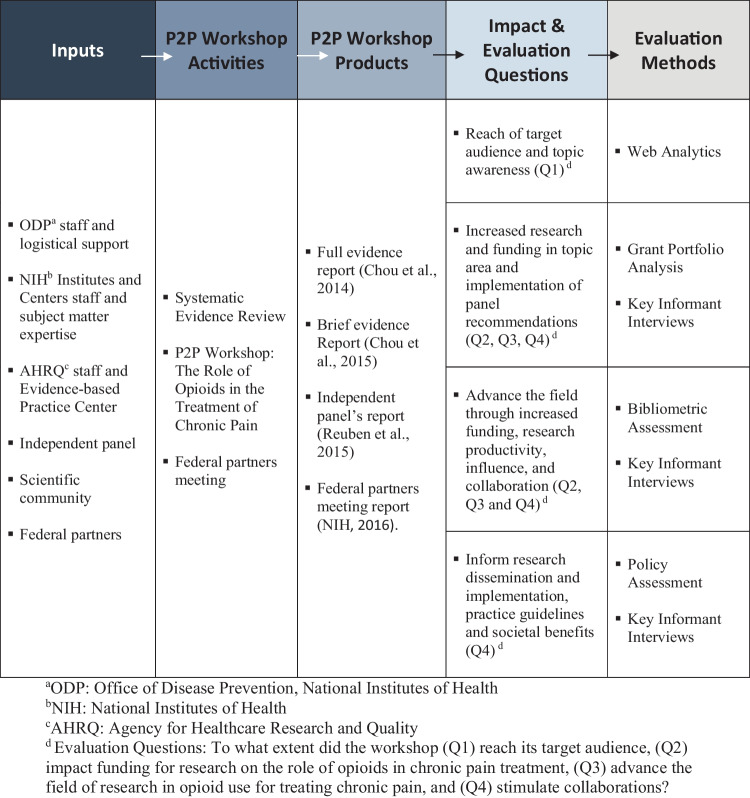
Pathways to Prevention (P2P) program evaluation framework

We developed an evaluation framework and assessment plan to examine the outcomes and impact of the Opioids P2P workshop. The evaluation framework (Fig. 1) outlined how the workshop’s inputs, activities, and products informed the assessment of outcomes and impact. Resources from NIH, ODP, and external partners (i.e., inputs) contributed to the workshop design and activities. The workshop generated four reports (i.e., products). The evaluation framework incorporated the four evaluation questions and informed our choice of a mixed methods approach with five components (i.e., web analytics, bibliometric assessment, grant portfolio analysis, policy assessment, and key informant interviews) for appraising outcomes and impact. Each method is described below.

### Web Analytics

ODP maintains extensive resources for the P2P program on its website (i.e., prevention.nih.gov). We used Google Analytics to track traffic on and engagement with the Opioids P2P workshop section of the website. We filtered the search to include reports containing the term “opioids” and exclude terms like “search” and “news,” which indicated opioids-related site content not directly relevant to the workshop. We exported all available traffic, event, and user demographic data from May 1, 2014 (date the workshop was announced) through September 30, 2021. Irrelevant pages, events and error messages were removed from the exported reports, which were then compiled into summary tables. Similar Google Analytics data for page traffic were obtained from AHRQ’s website, where the full evidence report is posted. We also retrieved counts of live and on-demand views of workshop recordings from the NIH VideoCast website (i.e., videocast.nih.gov).

### Bibliometric Assessment

We searched in the PubMed and Web of Science databases for the same time period for all citations in the scientific literature of the workshop’s three primary reports. The federal partners meeting report was excluded because it does not have a PubMed identification number. We obtained the total number of citations and the relative citation ratio (RCR) data for the identified articles using *iSearch: Publications*, an internal NIH Office of Portfolio Analysis tool. The RCR is a measure that quantifies a research article’s influence by using its co-citation network to field-normalize the number of citations it has received (Hutchins et al., 2016). An RCR score of 1.0 reflects the average scientific influence of NIH-supported articles in the same field and publication year. Scores < 1.0 indicate lower influence while those > 1.0 indicate higher influence. An additional impact measure, the Altmetric Attention score, was obtained using the Altmetric Explorer Tool (https://www.altmetric.com/). This score is a weighted count of all mentions (i.e., news, blogs, Facebook, and Twitter posts) tracked for individual research outputs. It serves as an indicator of the amount and reach of the attention an item has received (Altmetric, 2021).

We also assessed publication categories and funding sources for each article. Articles were classified into one of five mutually exclusive categories: *Scientific Article, Review Article, Commentary/Editorial/Position Paper, Practice Guideline,* and *Other*. The sources of funding identified in the articles were classified by organization type: *NIH, other U.S. government agency*, *international government, industry* (i.e., pharmaceutical, technology, diagnostic, health insurance and consulting companies, for-profit medical groups, and other businesses), *U.S. non-governmental organization (NGO)* (i.e., U.S. charitable foundations and nonprofit organizations, health systems, hospitals, and postsecondary academic institutions), *international NGO,* and *no support/missing* (i.e., the authors declared no funding was received or the publication did not list any sources of support). Data were analyzed using Stata SE 17.0.

### Portfolio Analysis

**Fig. 2:**
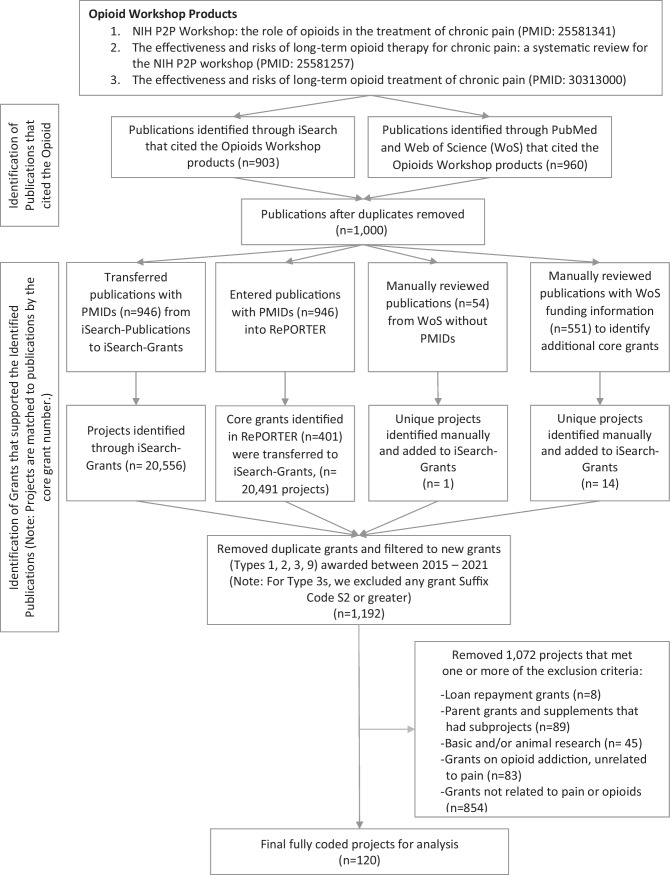
PRISMA diagram of portfolio analysis methodology

Using the PubMed identification numbers from publications retrieved in the bibliometrics analysis (*n* = 1000), we identified cited grant numbers in *iSearch: Publications*. We then used *iSearch: Grants* and NIH RePORTER (i.e., https://reporter.nih.gov/) to obtain detailed information about the supporting grants. We assessed Web of Science funding information to identify grant support for all publications lacking a PubMed identification number. We also searched for grants funded from January 2015 through September 2021 across all funding organizations represented in *iSearch*. We limited our search to newly awarded, competing grants and supplemental applications (NIH grant types: 1 = new application, 2 = renewal application, 3 = supplemental application (first year), and 9 = change of Institute at renewal) (*N* = 1192) and excluded projects that were not related to opioids or pain. In total, 120 projects met our inclusion criteria (see Fig. 2 for PRISMA diagram).

In collaboration with two NIH subject matter experts and informed by the workshop’s systematic evidence review and independent panel’s recommendations, we developed and tested a coding schema for the grant portfolio. The schema included 10 categories and 127 variables for characterizing study participants, research methods and study designs, outcomes, and type of pain studied (see online Appendix 1 for Coding Schema). Four trained coders from Westat manually applied the coding schema to each grant’s title, abstract, and public health relevance description. Two coders reviewed each grant and used *iSearch: Grants* to independently conduct their reviews and record their responses. For the 120 projects that met our inclusion criteria, ODP validated all project coding. ODP also reviewed a random selection of 15 percent of projects excluded from the analysis. Microsoft 365 Excel was used to produce descriptive statistics for the coded portfolio.

### Policy Assessment

We examined legislative tracking services and analytic metrics to identify policies—including practice guidelines and legislation—that may have been informed by the Opioids P2P workshop. We identified keywords from the workshop and report titles—such as “opioids,” “chronic pain,” “Pathways to Prevention,” and “National Institutes of Health”—and used them to search four databases from 2014 through 2021: Congress.gov, Congressional Quarterly, Bloomberg Government, and PlumX Metrics. We searched Congress.gov to identify any congressional activities related to the workshop topic, and the subscription-based Congressional Quarterly and Bloomberg Government services to identify guidelines and legislation that may have been informed by the workshop. PlumX Metrics was used to obtain information about online engagement with and references to the workshop reports, including research, policy, and practice guideline citations.

Each record identified in the database search was reviewed for relevance to the workshop and categorized as either *legislation* (i.e., enacted law) or *guideline* (i.e., general recommendations issued or used by an organization to support policies, standards, or procedures for routine, sound practice). Unrelated records were excluded, and all results were summarized in Excel.

### Key Informant Interviews

ODP staff partnered with evaluators from Westat to conduct in-depth interviews with several workshop key informants (*n* = 18). Interview goals were to better understand the potential influence of the workshop on research funding, post-workshop collaborations, and overall advancement of the opioids/chronic pain research field. ODP staff and NIH subject matter experts identified and invited the key informants to participate in interviews. Key informants included workshop participants such as NIH and other federal staff, workshop speakers, content area experts who contributed to workshop planning, and an investigator who conducted the systematic evidence review. In consultation with the NIH subject matter experts, ODP staff and Westat evaluators applied the evaluation framework to develop a 12-question interview protocol. The protocol was reviewed and approved by the Westat Institutional Review Board. Questions asked of key informants were open-ended; topics addressed included whether products from the Opioids P2P workshop informed the development of policies, guidelines, or legislation related to opioids and chronic pain; how the workshop and its products contributed to the research agenda and funding for opioids and pain research; and the extent to which the workshop stimulated new collaborations around opioids and chronic pain. The complete protocol is provided in the online Appendix 2. Westat evaluators conducted individual or small group interviews through a video conferencing platform (i.e., Zoom) from October 26 to December 6, 2021. All interview sessions were recorded, transcribed, coded, and imported into NVivo 11 for theme-based content analysis.

## Results

### Web Analytics

A total of 649 people registered for the Opioids P2P workshop. Although most registrants reported that they were health care professionals or researchers, the workshop attracted a wide variety of other individuals including advocates and administrators (see Fig. S5). Among those who registered to attend in-person, 44% were staff from federal agencies (66% of these were NIH staff), 17% were representatives from health care, 9% were from academia, 7% were from industry, and 12% reported some other affiliation. Nearly 450 people attended the 1 ½-day event in-person, while the workshop’s live NIH VideoCast received 792 views.

Workshop resources posted on the ODP website include the agenda, brief and full evidence reports, independent panel’s report, and federal partners meeting report. Webpages received 43,573 unique pageviews from the time the workshop was announced on May 1, 2014 through September 30, 2021. Since 2016, when download records first became available, workshop resources have been downloaded or opened 2096 unique times (see Table S4). The independent panel’s report was the most frequently downloaded resource. Archived recordings of the workshop were viewed 788 times since they were posted in October 2014. The brief and full evidence reports available on the AHRQ website were accessed 895 times.

### Bibliometric Assessment

**Fig. 3:**
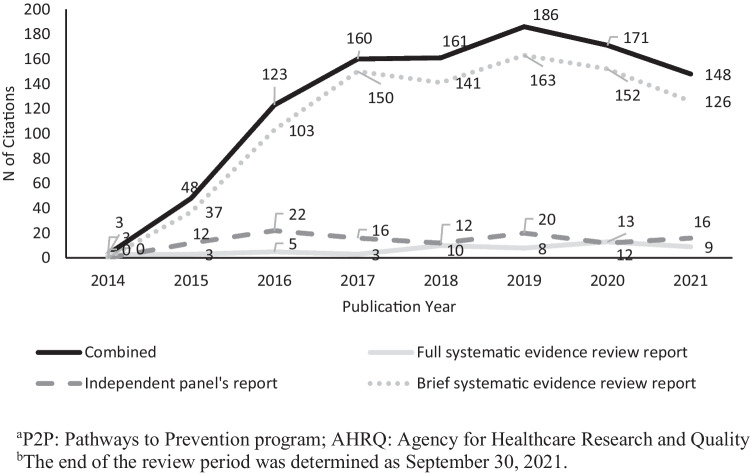
Number of citations per year of Opioids P2P ^a^ workshop primary reports, 2014–2021^b^

We identified 1000 journal articles citing at least one of the three primary workshop reports. Of these articles, 872 (87.2%) cited the brief evidence report, 110 (11.0%) cited the independent panel’s report, and 54 (5.4%) cited the full evidence report. Time trends demonstrate that the reports were cited frequently in the years following publication, with the greatest number of citations occurring in 2019 (see Fig. 2). Most citations were identified in scientific articles (58.6%); the remainder were in review articles (24.0%), commentaries/editorials/position papers (12.1%), practice guidelines (1.1%), or other article types (4.2%) (see Table 1 and Fig. 3).

**Table 1:** Number of citations of the Opioids P2P^a^ workshop primary reports by funding sources and publication types

**Funding source**	**Scientific article (*n* = 586)**	**Review article (*n* = 240)**	**Commentary/editorial (*n* = 121)**	**Other type (*n* = 42)**	**Practice guideline (*n* = 11)**	**Total (*N* = 1000)**
NIH^b^ (*n* = 250)	30.0%	21.3%	11.6%	16.7%	18.2%	25.0%
International Government (*n* = 182)	21.5%	17.9%	5.8%	11.9%	9.1%	18.2%
Other U.S. Government (*n* = 130)	16.6%	8.3%	5.0%	7.1%	36.3%	13.0%
U.S. NGO^c^ (*n* = 114)	16.0%	4.6%	0.0%	14.3%	27.3%	11.4%
Industry (*n* = 111)	13.1%	12.1%	0.8%	9.5%	0.0%	11.1%
International NGO (*n* = 105)	12.8%	10.0%	1.7%	7.1%	9.1%	10.5%
None declared/missing (*n* = 365)	21.8%	48.3%	78.5%	54.8%	27.3%	36.5%
Total (*N* = 1000)	58.6%	24.0%	12.1%	4.2%	1.1%	100.0%

^a^*P2P* Pathways to Prevention program

^b^*NIH* National Institutes of Health

^c^*NGO* non-governmental organization

NIH was reported as the most frequent funder of the research described in the articles (25.0%). The next most frequent funders were international governments (18.2%), U.S. government agencies other than NIH (13.0%), U.S. NGOs (11.4%), industry (11.1%), and international NGOs (10.5%). Approximately 36.5% of articles did not declare a funding source or had missing funding information (see Table 1).

Citation impact scores for the three primary workshop reports are shown in Table 2. The RCRs for the brief evidence report (56.77), independent panel’s report (6.09), and full evidence report (2.68) indicate high scientific influence relative to the average NIH-funded article in the same field and publication year (100.0%, 95.1%, and 82.9% percentile, respectively). Altmetric scores for the brief evidence report (563) and the independent panel’s report (312) are in the top 5% of most influential research documents tracked in the Altmetric database. At the end of the evaluation period, 679 of the 1000 citing articles had an RCR score available in *iSearch: Publications*. The average RCRs of the articles citing the three primary workshop reports were similar: 3.28 for articles citing the brief evidence report, 3.24 for articles citing the independent panel’s report, and 3.25 for articles citing the full evidence report. These metrics indicate that the citing articles were over three times more influential than the average NIH-supported article in the article’s field of study.

**Table 2:** Impact scores for the primary reports from the Opioids P2P^a^ workshop

Workshop published report	RCR^b^	Citations^c^	Average RCR^d^ of citations	Altmetric score
Independent panel’s report (Reuben et al., 2015)	6.09	110	3.24	312
Brief evidence report (Chou et al., 2015)	56.77	872	3.28	563
Full evidence report (Chou et al., 2014)	2.68	54	3.25	19

^a^*P2P* Pathways to Prevention program

^b^*RCR* relative citation ratio

^c^Citation counts sum to more than the number of citing articles analyzed (*N* = 1000) due to articles citing more than one product

^d^At the time of data collection, 679 projects had an RCR score available in iSearch-Publications

### Portfolio Analysis

We identified 120 projects that were funded by federal agencies after the workshop took place, cited one of the three primary workshop reports, and were directly relevant to the workshop’s topic of opioids and chronic pain. Of these projects, 110 (91.7%) were funded by NIH, eight (6.7%) by the U.S. Department of Veterans Affairs (VA), and two (1.7%) by the Centers for Disease Control and Prevention (CDC). The number of projects increased between fiscal years 2015 and 2017 (maximum = 27) and declined to the lowest count in 2021 (*n* = 8).

**Fig. 4:**
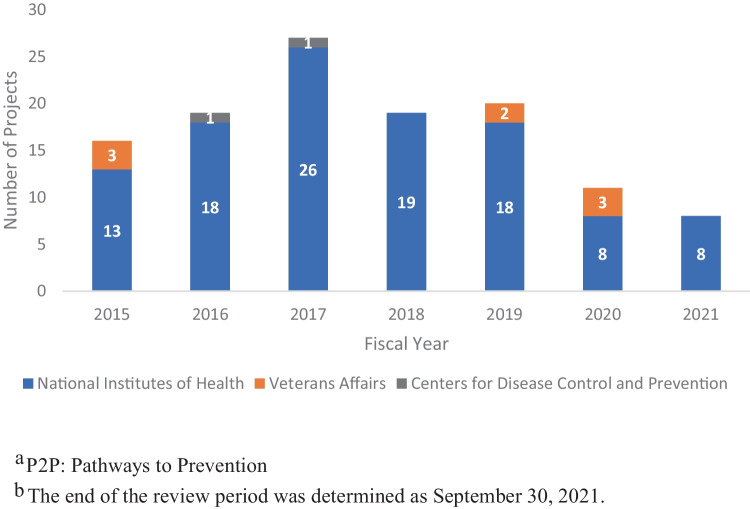
Number of grant-funded projects per year that cited the Opioids P2P ^a^ workshop primary reports, by Federal Agency Funder (2015–2021^b^)

Among the 120 relevant projects, 81 (67.5%) focused on people with pain receiving opioid or non-opioid therapy, including six (5.0%) focused on people with pain considering or within six months of starting pain therapy, and 20 (16.7%) focused on people receiving therapy of six months or longer. Ten (8.3%) projects focused on people with pain who were addicted to opioids. Twenty-eight (23.3%) projects were studying health care providers or systems of care. An additional 30 (25.0%) projects did not relate to one of these categories or focused on research training and career development. Most projects focused on chronic pain (*n* = 69, 57.5%), while 11 (9.2%) addressed acute pain, and 47 (39.2%) did not specify the pain type being studied or were training grants (see Table 3 for study participants and pain types and Fig. 4).

**Table 3:** Grant-funded projects citing the Opioids P2P^a^ workshop primary reports by project characteristics (2015–2021)

**Project characteristics**	**Number of projects (%)**^**b**^
**Study participant type**	
People with pain and on opioid or other pain therapy	81 (67.5%)
People with pain and length of therapy unclear	55 (45.8%)
People with pain on long-term (> 6 months) opioid therapy	20 (16.7%)
People with pain considering and/or starting opioid or other pain therapy and/or within the first 6 months of opioid therapy	6 (5.0%)
Health care provider/system of care	28 (23.3%)
People with pain and addiction to opioids	10 (8.3%)
Study participants not specified/other (includes training and career development projects)	30 (25.0%)
**Pain types studied**	
Chronic	69 (57.5%)
Acute/subacute	11 (9.2%)
Other/pain type not specified	47 (39.2%)
**Study design**	
Observational and population studies	57 (47.5%)
Intervention studies	42 (35.0%)
Randomized intervention studies	30 (25.0%)
Non-randomized intervention studies	6 (5.0%)
Intervention randomization unclear	6 (5.0%)
Pilot/feasibility/proof-of-concept/safety/planning grant	28 (23.3%)
Implementation science	24 (20.0%)
Methods research	8 (6.7%)
Patient registries	1 (0.8%)
Other or unclear study design	40 (33.3%)

^a^*P2P* Pathways to Prevention

^b^Project counts sum more than the number of projects analyzed (*N* = 120) due to projects relating to more than one topic

Nearly one-half of the projects were observational studies (*n* = 57, 47.5%). Of the 42 (35.0%) intervention studies, 30 (71.4%) were randomized trials and six (14.3%) were non-randomized; the randomization type was unclear for the remaining six projects (14.3%). There were also several pilot or feasibility studies (*n* = 28, 23.3%) and projects focusing on implementation science (*n* = 24, 20.0%). A few of the projects were methods development studies (*n* = 8, 6.7%). All study designs are shown in Table 3.

### Policy Assessment

We identified 13 policies issued after the workshop (i.e., 2016 through 2021) that cited or were likely influenced by the workshop’s independent panel or evidence reports. Seven policies (53.8%) were categorized as guidelines and six (46.2%) as legislation. Ten policies (76.9%) were developed by U.S. government agencies, one (7.7%) by state government (Pennsylvania), one (7.7%) by international government (Scotland), and one (7.7%) by an international NGO.

Four U.S. government agencies—CDC, VA, U.S. Department of Defense (DoD), and Centers for Medicare and Medicaid Services (CMS)—used information from the workshop’s independent panel and evidence reports to develop opioids and chronic pain-focused practice guidelines. The CDC’s guideline (Dowell et al., 2016) was developed for primary care providers, while the VA/DoD guideline (Department of Defense and Department of Veterans Affairs, 2017) was established for general use by clinicians throughout the VA and DoD healthcare systems. CMS developed two best practices guidelines: one for providers caring for Medicaid patients (Centers for Medicare & Medicaid Services, 2019), the other for states to help mitigate the opioid epidemic through non-opioid pain management options (Centers for Medicare & Medicaid Services, 2016). Legislation enacted by the U.S. Congress provided authority and funding for opioids and chronic pain research to the NIH and VA/DoD. Table S4 provides more detail on the individual policies and their sponsors.

### Key Informant Interviews

The 18 key informants who participated in structured interviews to discuss their perspectives on the workshop outcomes and impact identified several ways that the workshop contributed to the field of opioid use for treatment of chronic pain. Key informants noted that the workshop increased recognition of the problem of opioid overuse, misuse, and addiction and identified critical research gaps. The workshop was further perceived as successfully convening diverse perspectives on these issues. Several interviewees noted the importance of the systematic evidence review and its role in informing the 2016 CDC guidelines for prescribing opioids (Dowell et al., 2016). With regard to stimulating new research initiatives, the workshop’s influence on the subsequent development of the NIH’s Helping End Addiction Long-Term Initiative, or NIH HEAL Initiative^®^, launched in 2018 (https://heal.nih.gov/), was frequently mentioned. Increased support for research on managing pain with non-opioid approaches was another noted outcome. Additionally, the workshop was identified as contributing to several trans-agency collaborations, including the NIH-DoD-VA Pain Management Collaboratory and cited as the catalyst for two follow-on pain management conferences sponsored by the VA. Table S5 in the online Appendix l liststs selected guidelines, initiatives, and other activities identified by interviewees as influential outcomes of the workshop.

## Discussion

P2P is a unique NIH program in which scientific workshops on high-priority topics have the potential for broad impact on health research, policy, and practice. By systematically reviewing available evidence, analyzing research portfolios, obtaining public input, and bringing together thought leaders and federal agency partners for discussion and deliberation, the P2P program gathers and disseminates comprehensive, leading-edge knowledge in each topic area considered. In particular, the program’s use of an independent panel to review, synthesize, and propose next steps contributes to appraisal of a public health issue that is as unbiased as possible.

Unbiased appraisal was especially needed at the time of the Opioids P2P workshop. When workshop planning was initiated in 2012, there was growing concern that harms associated with prescription opioid use were reaching crisis levels in the U.S. By the time the workshop took place in 2014, opioid addiction and misuse had become a major public health problem, and it was not clear how best to address or mitigate it. Reducing opioid prescribing was one possible solution, but with over 100 million Americans experiencing chronic pain (Institute of Medicine, 2011), restricting access to a powerful pain treatment could cause suffering and motivate some individuals to seek heroin or other street drugs for relief. There was disagreement among pain researchers and clinicians, with some arguing that opioids were safe and effective if used as prescribed, and others insisting that they were highly addictive and not appropriate in medical practice. The resulting confusion and frustration among clinicians, researchers, and the public indicated a sizable gap between scientific knowledge and informed practice.

The workshop was well-suited for building consensus and identifying unbiased potential solutions. Our assessment of its impact, guided by an evaluation framework and focused questions, was multicomponent, incorporated five distinct methods, and included both quantitative and qualitative data. It also incorporated a policy assessment, a feature not typically employed in impact assessments of health research programs. Our study showed that the workshop attracted a broad audience of clinicians, researchers, advocates, and others, from federal agencies, healthcare organizations, industry, and academia. Its published reports, cited one thousand times during our seven-year study period, have had high impact in the scientific literature and continue to be referenced several years after the workshop took place. The workshop helped foster development of over one hundred new research projects supported through grants funded by three federal agencies. It also informed several pieces of national legislation and guidelines from influential organizations. Key informant interviews further highlighted the workshop’s influence on development of the NIH HEAL Initiative® and multi-agency collaborations, especially the NIH-DoD-VA Pain Management Collaboratory.

Our assessment showed that the workshop and follow-up activities identified research gaps and stimulated increased funding and federal-level support for opioids and chronic pain research. In particular, the workshop guided research plans and priorities at the NIH. Following the workshop, NIH Pain Consortium members published a paper proposing that the prescription opioid and pain crises should be addressed jointly, rather than focusing primarily on opioid misuse with insufficient attention to the needs of people suffering from chronic pain (Thomas et al., 2015). NIH operationalized the recommended approach, as exemplified by the NIH HEAL Initiative^®^, which supports over 1000 research projects in every state with the aim of improving pain management and the prevention and treatment of opioid use disorder and addiction.

In terms of clinical practice, the workshop served as a key resource for the CDC in its development of new guidelines for the use of opioids in treating chronic pain (Dowell et al., 2016). The CDC guidelines have been broadly disseminated and widely adopted in the U.S and internationally. In 2022, opioid prescribing continued to decline (Centers for Disease Control & Prevention, 2021), and deaths from prescription opioid overdoses may be leveling off (Hedegaard et al., 2021).

### Limitations

There are several limitations to our assessment of the Opioids P2P workshop’s impact. Information about virtual participants was limited. Affiliation and profession information was collected only for in-person registrants, and VideoCast only reports the total number of live views, which may overcount participants who left and returned to the VideoCast. Our bibliometric analysis focused on number of citations, and RCR and Altmetric scores to assess impact of the workshop’s primary reports; inclusion of additional measures may have provided a more comprehensive picture of impact. The list of projects examined in the portfolio analysis was generated from grant numbers specified in publications identified in the bibliometric analysis; some projects may be missing if funding was not properly attributed, or the workshop products were not appropriately referenced. The policy assessment relied on a defined set of search terms and four online resources; inclusion of other search terms and resources may have identified additional policies informed by the workshop. Key informant interviews were conducted approximately seven years after the workshop tool place; accuracy of interviewees’ recall of events and outcomes may have lessened over time. Finally, results from the Opioids P2P workshop assessment may not be generalizable to other topics in the P2P program.

## Conclusions

The Opioids P2P workshop and follow-up activities have identified gaps in scientific knowledge, informed clinical practice, and catalyzed change on a national level for addressing the prescription opioid crisis. We conclude that the workshop had an impact on the field and was influential in the U.S. response to the opioid crisis. The P2P program continues to focus on timely and important public health issues; future work will be needed to assess the impact of other P2P workshops.

## Supplementary Information

Below is the link to the electronic supplementary material.

Supplementary file1 (DOCX 145 KB)

## Data Availability

Data available from the corresponding author on reasonable request.
