# Supplementary material for: Opioids and Chronic Pain: Impact of the NIH Pathways to Prevention Evidence-Based Workshop Program
Source: Prev Sci. 2023 Jul 15;24(6):1091–101. doi: 10.1007/s11121-023-01563-9 (PMC10423705; doi:10.1007/s11121-023-01563-9)
Supplement: Supplementary file 1 — Supplementary file1 (DOCX 145 KB) [file 11121_2023_1563_MOESM1_ESM.docx]

**APPENDIX TO *PREVENTION SCIENCE* ARTICLE**

Opioids and Chronic Pain: Impact of the NIH Pathways to Prevention Evidence-Based Workshop Program

Carrie N. Klabunde^1^, Liberty Walton^1^, Keisha L. Shropshire^1^, Luis F. Ganoza^1^, Jen Hession^1^, Kat Schwartz^1^, Elizabeth Vogt^1^, David A. Thomas^2^, Wendy B. Smith^3^, Melissa C. Green Parker^1^, and Charlene L. Liggins^1^

^1^Office of Disease Prevention, Office of the Director, National Institutes of Health, Bethesda, MD

^2^Office of Research on Women’s Health, Office of the Director, National Institutes of Health, Bethesda, MD

^3^Office of Behavioral and Social Sciences Research, Officer of the Director, National Institutes of Health, Bethesda, MD

Appendix content: Portfolio Analysis Coding Scheme; Key Informant Interview Protocol; Web Analytics and Key Informant Interview Results

**Appendix Section 1**.

***Portfolio Analysis Coding Schema***

# **A. Exclusion Criteria**

1. Basic, molecular, cellular, and/or basic pharmaceutical science related to opioids, pain, or pain treatment
2. Animal Models related to opioids, pain, or pain treatment
3. Human Tissue related to opioids, pain, or pain treatment (except for genetic studies associated with methods research to assess risk for opioid abuse)
4. Patients with addiction, depression, or other mental health conditions AND no mention of pain as an outcome (except for genetic studies associated with methods research to assess risk for opioid abuse)
5. Intravenous or intramuscular administration of opioids outside of a clinical setting
6. Tramadol only
7. Other (no mention of either pain, pain treatment, or opioids)

# **B. Study Participants (Focus of the research assessments, measures, and outcomes)**

1. Persons with Pain and on opioid or other pain therapy (Note: If opioid treatment length is unspecified, select only B.1. If specified, select B1 and either B1a. and/or B1b.)

1a. Persons with Pain considering and/or starting opioid or other pain therapy and/or within the first 6 months of opioid therapy (as part of clinical care or an intervention)

1b. Persons with Pain on long-term (>6 months) opioid therapy

1. Persons with Pain with addiction to opioids
2. Health care provider/system of care
3. Study Participants not specified/Other (could include training and career development)

# **C. Pain Types**

1. Chronic Pain (e.g., persistent, intractable, refractory)
2. Acute/Subacute
3. Other/Pain type not specified (could include projects related to physician prescribing behavior or training programs)

# **D. Study design**

## Pilot/feasibility/proof-of-concept/safety/planning grant (can be double-coded with other study designs)

## Methods research

- 1. Validation of **existing** patient assessment tools **to assess pain and/or risks for opioid misuse, overdose, abuse; to assess long-term outcomes of opioid use**
  2. Development of **new** patient assessment tools **to assess pain and/or risks for opioid misuse, overdose, abuse; to assess long-term outcomes of opioid use**
  3. Assess/validate **use of pharmacogenetics** and biomarkers as part of patient assessment tools

## Observational and population studies (select a-d if developing new cohorts/questionnaires/surveys)

- 1. Longitudinal cohorts
  2. Cross-sectional cohort
  3. Questionnaires/Surveys/Interviews
  4. Electronic Health Records, Pharmacy Records, and Insurance claims data
  5. Analysis of existing data (also double-code with D.3.a, D.3.b, D.3.c, or D.3.d)

## Intervention Studies

1. Non-randomized intervention studies
2. Randomized intervention studies
3. Analysis of longer-term follow-up cohort data from existing intervention studies

##

## Implementation Science - cost-benefit or budget analysis, uptake of clinical guidelines/policies, barriers or facilitators to dissemination and implementation (double code with 4. Intervention Studies if pragmatic trial)

## Patient Registries – building registries of people with pain or opioid treatment for pain

## Other or unclear (e.g., Infrastructure, training, career development awards)

# **E.** **Methods – Patient Assessment Tools**

For **testing/validating** **existing** patient and risk assessment tools

1. NIH Patient Reported Outcomes Measurement Information System (PROMIS)
2. The Pain Assessment Screening Tools and Outcomes Registry (PASTOR)
3. The Collaborative Health Outcomes Information Registry
4. The Orafacial Pain: Prospective Evaluation and risk Assessment (OPERA)
5. The Multidisciplinary Approach to the Study of Chronic Pelvic Pain (MAPP)
6. PhenX
7. Screener and Opioid Assessment for Patients in Pain (SOAPP)
8. Other screening tools – please specify
9. Specify “Other” tools

# **F. Intervention Study Components - Effective/Comparative effectiveness of opioids and other therapies when seeking to impact/improve pain, functioning and/or “Quality of Life”**

1. Opioids vs. Placebo
2. Opioids vs. Opioids (includes dosing/tapering)
3. Opioids vs no-Opioids or placebo (for cessation of opioid use)
4. Opioids vs. Non-opioid pain interventions (pharmacological or non-pharmacological)
5. Opioids + Non-Opioid pain interventions (pharmacological or non-pharmacological)
6. Non-pharmacologic Interventions or components, (Specify types a-f) –
   1. Physical therapy
   2. Behavioral therapy,
   3. Mindfulness training
   4. Hypnosis
   5. Acupuncture
   6. Other
7. Other

# **G1.** **Patient (Individual)-Level Risk Mitigation Methods and Strategies – seeking to alleviate risky behavior, opioid abuse, misuse, and/or addiction**

- 1. Dosing/Tapering Strategies
  2. Opioid management plans
  3. Patient agreement
  4. Patient education
  5. Urine drug screening
  6. Monitoring instruments
  7. Frequent monitoring intervals
  8. Pill counts
  9. Access and/or Use of Naloxone
  10. Other

# **G2. Provider(s)(system)-Level Risk Mitigation Methods and Strategies – seeking to alleviate risky behavior, opioid abuse, misuse, and/or addiction**

1. Educate or Assess physician behavior or prescribing practices (including prescribing Abuse-deterrent drug formulations)
2. Multi-disciplinary team approach to care/care coordination
3. Electronic Health Records (E.H.R), Pharmacy Records – including prescription monitoring program data; integration of **patient risk assessment tools**; monitor **harms and adverse side effects** of opioid use
4. Development of other clinical tools to monitor patient outcomes for use in care settings (i.e., menu or decision-support prompts to inform provider behavior)
5. Reimbursement or payment alternatives/incentive models for care coordination and non-pharmacologic or other opioid alternatives
6. Other

# **H.** **Covariates, Measures, and Outcomes of interest (when assessing study aims)**

1. Pain levels (intensity and length of pain experience)
2. Opioid use
3. Reduced doses or cessation of opioids (could include provider prescribing behavior or intervention)
4. Risk or occurrence of opioid misuse, abuse, addiction, overdose, or risky behavior
5. Withdrawal symptoms
6. Psychosocial factors (e.g., stress, loneliness, social disruption)
7. Psychological harms (mental health, anxiety, depression, emotions)
8. “Quality of life” (exact term)
9. Physical or motor functioning or functional status
10. Cognitive harms, effects, function
11. Patient demographics (age, race, ethnicity)
12. Patient co-morbidities (including past or current alcohol disorders, substance use disorders, mental disorders, medical comorbidities, and high risk for addiction)
13. Gastrointestinal-related harms
14. Falls
15. Fractures
16. Motor vehicle accidents
17. Endocrinological harms/sexual dysfunction
18. New Infections
19. Cardiovascular events
20. Sleep
21. Utilization of health care (e.g. Increase in ER/ED visits, care retention)
22. Institution/Provider Level Factors
23. Other (e.g., mortality, unspecified clinical outcomes) – please specify
24. Specify ‘Other’ Outcome:

#

# **I.** **Study Setting**

1. Clinical Setting
   1. Outpatient settings (e.g., primary care, pain clinics, addiction clinics, other specialty clinics home health care)
   2. Inpatient (hospital, addiction treatment settings, nursing home)
2. Non-clinical setting/Research setting/Laboratory
3. Other or unclear

# **J.** **Study population**

## Age Group

- 1. Children (under 18)
  2. Young Adults (Ages 18-30)
  3. Middle-aged Adults (Ages 31-64)
  4. Older Adults (Ages 65 and older, Nursing Home residents, elderly, geriatric)
  5. Age Group – Adults (age unspecified)
  6. Other/Unclear

## Sex/Gender

1. Male
2. Female
3. Trans-Men/Women
4. Other/Unclear

## Race/Ethnicity

1. American Indian or Alaska Native
2. Asian
3. Black or African American
4. Hispanic/Latino
5. Native Hawaiian or Other Pacific Islander
6. White
7. “Multi-racial”/“Multi-ethnic” (non-specified)
8. Other/Unclear

## Special Populations

1. Incarcerated/institutionalized/Recently paroled (including nursing home residents)
2. Sexual or gender minorities
3. Low income, poverty, under-resourced (including Medicaid users, uninsured, homeless)
4. Military/veterans
5. People with disabilities
6. Pregnant/post-partum persons
7. Urban
8. Rural
9. Other– please specify
10. No special population
11. Specify ‘Other’ Special Populations

**Appendix Section 2.**

***Key Informant Interview Protocol***

**Pathways to Prevention Workshop:**

***The Role of Opioids in the Treatment of Chronic Pain, 2014***

**Impact Assessment Interview Guide**

Thank you again for your willingness to participate in this interview. My name is ______, and I work for Westat. I’ll be conducting the interview today on behalf of the NIH Office of Disease Prevention.

As described in the email about the interview, the NIH Office of Disease Prevention is interested in learning about the impacts of the Pathways to Prevention, or P2P, Program by examining the impacts of specific P2P workshops. For this interview, we’re focusing on the 2014 workshop entitled *The Role of Opioids in the Treatment of Chronic Pain*.

**INFORMED CONSENT**

Before we begin, I want to inform you that your participation in this interview is voluntary. You can choose not to answer any question and stop the interview at any time. The feedback you provide is very important and will be used to make improvements in the P2P program, so it’s important that you speak candidly about your experiences and opinions about the workshop and its products (systematic evidence review, panel recommendations, and Federal Partners Meeting report) as well as your thoughts on the implementation of panel recommendations. In the assessment products, responses will be aggregated, and your name will not be attributed to interview responses.

The interview will take approximately 30 minutes, and with your permission, I would like to record our discussion so that I can concentrate on what you are saying and will not have to take detailed notes. The recording will be destroyed once the project is over. Do I have your permission to record this interview? Do you have any questions before we get started?

**Advancement of the Opioid Chronic Pain Research Field**

1. We understand that you were involved in the Opioid P2P process as [*insert roles/responsibilities*]? Is this correct?
   1. PROBE: did you attend the workshop? If so, how did you participate?
   2. PROBE: Did you attend the Content Area Experts meeting prior to the workshop? If so, how did you participate?
   3. PROBE: Did you attend the Federal Partners meeting after the workshop? If so, how did you participate?
2. [ODP/IC Staff Only] I want to start by asking a question about workshop products, panel recommendations, the systematic evidence review, and the federal partners meeting report. How were workshop products disseminated after the workshop? In your opinion, what were the most effective ways of disseminating these products to the targeted audiences?

There are many ways the P2P workshop and its products may have contributed to advancing the field of opioid use for treatment of chronic pain, both directly and indirectly. [*For participants other than ODP/IC staff, add:* “These products include the panel recommendations, systematic evidence review, and federal partners meeting report.”] One example is contributing to the development of new clinical guidance or policies. For example, P2P products such as the evidence review and panel report paper were cited in the 2016 C*DC Guideline for Prescribing Opioids for Chronic Pain*.

1. Based on your knowledge, are there other examples where P2P workshop products informed development of policies or guidelines related to opioids and pain?
2. Have they informed development of legislation or congressional activities related to opioids and pain?
3. Are you aware of other tangible benefits that resulted from the workshop or its products, such as new data sources, or informing opioid and pain-related trainings, conferences, workshops, practice guidelines, or other educational opportunities?
4. How did the P2P workshop and its products contribute to the research agenda and funding for opioid and pain research at NIH and other agencies?
   1. PROBE: Did it contribute to or inform the NIH HEAL initiative?
   2. PROBE: Did it contribute to or inform research priorities?
   3. PROBE: Did it contribute to or inform any other new funding opportunity announcements?
5. The P2P panel had recommendations about increasing research in specific areas. Are you aware of an increase in research and/or funding for:
   1. Studies or initiatives examining types of pain, diseases, and patients most likely to benefit or incur harm from opioid use?
   2. Research to develop and evaluate multidisciplinary pain interventions?
   3. The development of measurement tools that identify patient risk and outcomes related to dose effect and side effects of long-term opioid use?
   4. Research and/or quality improvement efforts to increase evidence-based decision making?
   5. PROBE: Does the HEAL initiative address any of these areas? IF YES: Can you describe?
   6. PROBE: Has PCORI-funded opioids research in any of these areas? IF YES: Can you describe?

**Collaborations Resulting from Opioid/Chronic Pain P2P**

Now I want to ask you about collaborations that may have resulted from the workshop.

1. To what extent do you think the P2P workshop facilitated new collaborations around opioids and pain? Are there any specific examples of collaborations that you can identify that resulted from the P2P workshop?

PROBE on PANEL RECOMMENDATIONS FOR COLLABORATIONS:

- Were there any collaborations around expanding pain registries or standardizing assessment tools? Can you provide examples?
- Co-sponsorship of conferences or workshops that brought together scientists and clinicians or multidisciplinary teams to help guide research priorities and identify gaps? Can you provide examples?
- Pain-research focused collaborations, such as the NIH Pain consortium? Can you provide examples?

1. [ODP/IC Staff Only] To your knowledge, have ODP and the sponsoring ICs (NIDA, NINDS, NIH Pain Consortium) continued to work together in the area of opioids and pain as a result of the workshop? Can you provide examples?
   1. PROBE: IF YES: How have they continued to collaborate?

**Implementation of Panel Recommendations**

The P2P panel report included several recommendations. I hope you had a chance to review them, but I’ll also display them on the screen now.

1. For which recommendations has significant progress been made?
   1. PROBE: In what ways can progress be attributed to the P2P workshop?
   2. PROBE: [If not addressed in previous responses, specifically ask about:]

- Rec #4: Electronic health record vendors and health systems should incorporate decision support for pain management…
- Rec #5: Researchers on the effectiveness and harm of opioids should consider alternative designs…
- Rec #8: In the absence of definitive evidence, clinicians and health care systems should follow current guidelines…
- Rec #9: NIH or other federal agencies should sponsor conferences to promote harmonization of guidelines…

1. Which recommendations, if any, are lagging in progress?

**Closing**

1. As a final question, do you have any suggestions for ways that the overall P2P program (or NIH) could increase the impact of the panel recommendations?

Are there ways to increase the reach of the panel recommendations so that they are more impactful?

What can the P2P Program (or NIH) do to ensure panel recommendations are coordinated with or inform other similar research efforts?

These are all the questions I have for you. Do you have any further comments or thoughts that you would like to share?

Thank you for providing your feedback.

**Appendix Figure 1.**

*Self-Reported Profession of Opioids Pathways to Prevention (P2P) Workshop Registrants (n=440 respondents)*


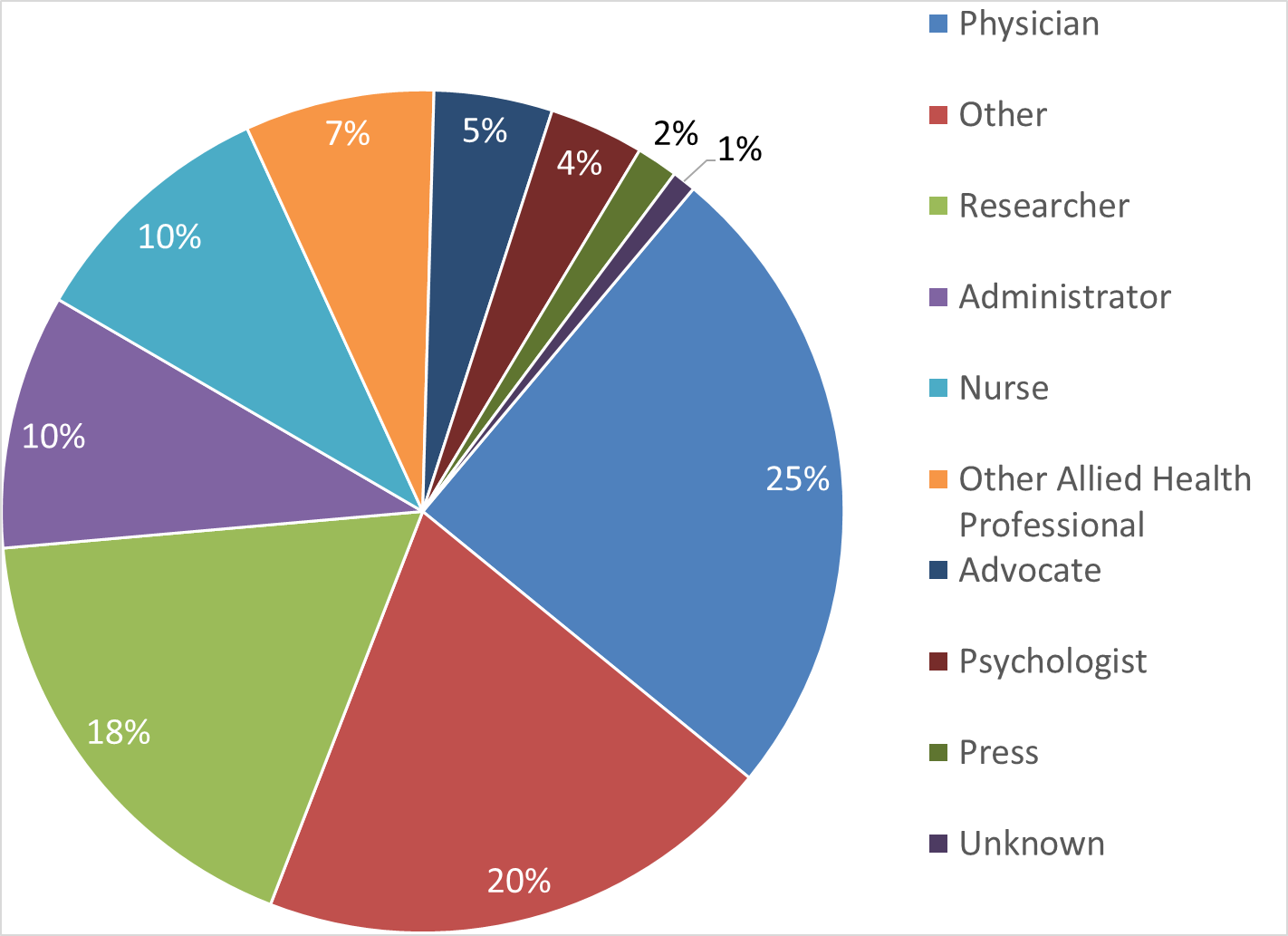


**Appendix Table 1.**

*Engagement with Opioids P2P^a^ Workshop Web-based Resources^b^ (2016-2021)*

| **Document** | **All Unique Clicks or Downloads** | **% of Total** |
| --- | --- | --- |
| Independent panel's report | 1149 | 54.8% |
| Opioids/chronic pain infographic | 217 | 10.4% |
| Full systematic evidence review report | 207 | 9.9% |
| Federal partners meeting report | 204 | 9.7% |
| Brief systematic evidence review report | 140 | 6.7% |
| Program book | 133 | 6.3% |
| Agenda | 46 | 2.2% |
| **Total** | **2096** |  |

^a^P2P: Pathways to Prevention program

^b^Web-based resources were accessed at: <https://prevention.nih.gov/research-priorities/research-needs-and-gaps/pathways-prevention/role-opioids-treatment-chronic-pain>

**Appendix Table 2.**

*Guidelines, Legislation, and Agency Initiatives Informed by the Opioids P2P^a^ Workshop*

| **Title (Sponsors, Year Issued or Implemented)** | **Brief Description (Data Source)** |
| --- | --- |
| **Guidelines** |  |
| [CDC Guideline for Prescribing Opioids for Chronic Pain—United States](https://www.ncbi.nlm.nih.gov/pmc/articles/PMC6390846/pdf/nihms-1008876.pdf) (CDC^a^, 2016) | Provides 12 recommendations for primary care clinicians in prescribing opioids to treat adult patients with chronic pain outside of active cancer treatment, palliative care, and end-of-life care. Cited brief SER^b^ and P2P panel report^c^ (Bibliometric Analysis) |
| [CMCS Informational Bulletin: Best Practices for Addressing Prescription Opioid Overdoses, Misuse and Addiction](https://www.medicaid.gov/sites/default/files/Federal-Policy-Guidance/Downloads/cib-02-02-16.pdf) (CMS^d^, 2016) | Describes Medicaid strategies for managing prescription opioids and preventing opioid-related harms. Cited brief SER (PlumX Metrics^e^) |
| [Prescribing Guidelines for Pennsylvania-Geriatric Pain: Opioid Use and Safe Prescribing](https://www.dos.pa.gov/ProfessionalLicensing/BoardsCommissions/Documents/PA%20Guidelines%20Geriatric%20Pain%20Treatment%20(2).pdf) (PAMED^f^, 2016) | Provides guidance for how health care providers can improve patient outcomes when providing opioid treatment, including avoiding potential adverse outcomes associated with the use of opioids to treat pain. Cited brief SER (PlumX Metrics) |
| [VA/DoD Clinical Practice Guideline for Opioid Therapy for Chronic Pain](https://www.healthquality.va.gov/guidelines/Pain/cot/VADoDOTCPG022717.pdf) (VA^g^, DoD^h^, 2017) | Provides practitioners throughout the VA and DoD healthcare with evidence-based recommendations and a framework by which to evaluate, treat, and manage the individual needs and preferences of patients with chronic pain who are on or being considered for long-term opioid therapy. Cited brief SER and P2P panel report (Congress.gov^i^) |
| [Management of Chronic Pain in Children and Young People: A National Clinical Guideline](https://www.gov.scot/binaries/content/documents/govscot/publications/advice-and-guidance/2018/03/management-chronic-pain-children-young-people/documents/00533194-pdf/00533194-pdf/govscot%3Adocument/00533194.pdf) (Scottish Government, 2018) | Summarizes the best available evidence and provides expert guidance on managing pediatric chronic pain in children and young people. Cited brief SER (PlumX Metrics) |
| [CMCS Informational Bulletin: Medicaid Strategies for Non-Opioid Pharmacologic and Non-Pharmacologic Chronic Pain Management](https://www.medicaid.gov/federal-policy-guidance/downloads/cib022219.pdf) (CMS, 2019) | Provides information to states seeking to promote non-opioid options for chronic pain management and Medicaid coverage options for states in response to the opioid epidemic. Supports the goal of reducing opioids use in pain management included in the President’s Initiative to Stop Opioid Abuse and Reduce Drug Supply and Demand and is consistent with the U.S. Department of Health and Human Service (HHS) 5-Point Strategy to Combat the Opioid Crisis. Cited brief SER (PlumX Metrics) |
| [SIGN 136 - Management of chronic pain: A national clinical guideline](https://www.sign.ac.uk/media/1108/sign136_2019.pdf) (Scottish Intercollegiate Guidelines Network - Healthcare Improvement Scotland, 2019) | Provides recommendations based on current evidence for best practice in assessing and managing adults with chronic non-malignant pain in non-specialist settings. Cited brief SER (PlumX Metrics) |
| **Legislation** |  |
| [S.524 – Comprehensive Addiction and Recovery Act of 2016](https://www.congress.gov/114/plaws/publ198/PLAW-114publ198.pdf) (Public Law 114-198; 114^th^ U.S. Congress, 2016) | Grants NIH authority to intensify and coordinate NIH research on the understanding of pain, therapies for chronic pain, and alternatives to opioids for pain treatments. The prioritization and direction of federally funded pain research must consider recommendations made by the IPRCC^j^. Cited NIH IPRCC. (Congress.gov) |
| [H.R.1625 - Consolidated Appropriations Act, 2018](https://www.congress.gov/bill/115th-congress/house-bill/1625) (Public Law 115-141; 115^th^ U.S. Congress, 2018) | Permits NIH to transfer funds specifically appropriated for opioid addiction, opioid alternatives, pain management, and addiction treatment to other NIH Institutes and Centers for the same purpose. Language supports and aligns with other legislation and guidelines that cite P2P publications. (Congress.gov) |
| [H.R.2810 - National Defense Authorization Act for Fiscal Year 2018](https://www.congress.gov/bill/115th-congress/house-bill/2810) (Public Law 115-91; 115^th^ U.S. Congress, 2018) | Directs the DoD to study the effectiveness of training provided to military health care providers on opioid prescribing practices, initiatives in opioid safety, the use of the VA/DoD Clinical Practice Guideline for Management of Opioid Therapy for Chronic Pain, and other related training. Cited VA/DoD Clinical Practice Guideline for Opioid Therapy for Chronic Pain, 2017 (Congress.gov) |
| [H.R.6 - Substance Use-Disorder Prevention that Promotes Opioid Recovery and Treatment for Patients and Communities Act (or the SUPPORT for Patients and Communities Act)](https://www.congress.gov/bill/115th-congress/house-bill/6) (Public Law 115-271; 115^th^ U.S. Congress, 2018) | Expands NIH’s unique research initiatives to include cutting-edge research that is urgently required to respond to a public health threat. Commissioned a report instructing the HHS Secretary to, among other things, consider incorporating relevant guidance from the VA/DoD Clinical Practice Guideline for Opioid Therapy for Chronic Pain into HHS guidance. Cited VA/DoD Clinical Practice Guideline for Opioid Therapy for Chronic Pain, 2017 (Congress.gov) |
| [H.R.1865 - Further Consolidated Appropriations Act](https://www.congress.gov/bill/116th-congress/house-bill/1865) (Public Law 116-94; 116^th^ U.S. Congress, 2020) | Grants NIH authority to transfer funds specifically appropriated for opioid addiction, opioid alternatives, pain management, and addiction treatment to other NIH Institutes and Centers for the same purpose. Directs funding to be used for activities authorized under section 8071 of the SUPPORT for Patients and Communities Act (Public Law 115–271), which cited VA/DoD Clinical Practice Guideline for Opioid Therapy for Chronic Pain, 2017. Cited VA/DoD Clinical Practice Guideline for Opioid Therapy for Chronic Pain, 2017 (Congress.gov) |
| [H.R.6395 - William M. (Mac) Thornberry National Defense Authorization Act for Fiscal Year 2021](https://www.congress.gov/bill/116th-congress/house-bill/6395/text) (Public Law 116-283; 116^th^ U.S. Congress, 2021) | Requires the Secretary of Defense to develop a policy and tracking mechanism to monitor and provide oversight of opioid prescribing to ensure medication prescribing practices among health professionals across the military health system conform with the VA/DoD clinical practice guidelines, and CDC and FDA prescribing guidelines. Cited VA/DoD Clinical Practice Guideline for Opioid Therapy for Chronic Pain, 2017 and CDC Guideline for Prescribing Opioids for Chronic Pain—United States, 2016 (Congress.gov) |
| **Agency Initiatives** |  |
| [PA:15-188: Developing the Therapeutic Potential of the Endocannabinoid System for Pain Treatment (R01)](https://grants.nih.gov/grants/guide/pa-files/PA-15-188.html) (7 NIH Institutes and Centers, 2015) | Funding opportunity announcement for research projects to develop the therapeutic potential of the endocannabinoid system (alternative to opioids) for pain treatment. (Key Informant Interviews) |
| National Pain Strategy (IPRCC, 2016) | Comprehensive report outlining a strategy for decreasing the prevalence of pain and its associated morbidity and disability across the lifespan. (Key Informant Interviews) |
| State-of-the-Art Conference: Non-pharmacological Approaches to Chronic Musculoskeletal Pain Management (VA, 2016) | Follow-on conference to the Opioids P2P workshop, convened by the VA, to reach consensus on policy recommendations to improve non-pharmacological approaches to chronic musculoskeletal management of pain and addiction and improve opioid safety. (Key Informant Interviews) |
| Pain Management Collaboratory (NIH, VA, DoD, 2017) | Collaboratory supported by a central coordinating center, comprising 11 [pragmatic clinical trials](https://painmanagementcollaboratory.org/pragmatic-studies/) studying nonpharmacological approaches for managing pain and common co-occurring conditions in military and veterans’ health care. (Key Informant Interviews) |
| [RFA-DA-18-012: Development of a Device to Objectively Measure Pain (R43/R44)](https://grants.nih.gov/grants/guide/rfa-files/RFA-DA-18-012.html) (NIH/NIDA, 2018) | Small Business Innovation Research initiative to develop a technology/device that objectively indicates the presence and level of pain. (Key Informant Interviews) |
| [Helping to End Addiction Long-term (HEAL) Initiative](http://heal.nih.gov/) (12 NIH Institutes and Centers, 2018) | Trans-NIH effort to improve prevention and treatment strategies for opioid misuse and addictions and to enhance pain management. (Key Informant Interviews) |
| Opioid Risk Evaluation and Mitigation Strategies (REMS) (FDA, 2018) | Required strategies for all opioids intended for outpatient use to ensure that benefits outweigh risks; intended to be used on the national- and state-level to reduce prescription opioids abuse, misuse, addiction, overdose, and deaths. (Key Informant Interviews) |
| [Pain Management Best Practices Inter-Agency Task Force Report](https://www.hhs.gov/opioids/prevention/pain-management-options/index.html) (HHS; DoD; VA; ONDCP^k^, 2019) | Report summarizing the findings and recommendations of the Task Force, charged with making recommendations for best practices in managing acute and chronic pain. (Key Informant Interviews) |
| [Overdose Data to Action Program](https://www.cdc.gov/drugoverdose/od2a/index.html) (CDC, 2019) | Program supporting state, territorial, county, and city health departments in collecting high quality, comprehensive, timely data on overdoses and in using the data to inform prevention and response efforts. (Key Informant Interviews) |
| State-of-the-Art Conference: Effective Management of Pain and Addiction: Strategies to Improve Opioid Safety (VA, 2019) | Follow-on conference to the Opioids P2P workshop, convened by the VA, to reach consensus on policy recommendations to improve management of pain and addiction and improve opioid safety. (Key Informant Interviews) |

^a^CDC: Centers for Disease Control and Prevention

^b^SER: Systematic Evidence Review (Chou et al., 2015)

^c^P2P panel report: Independent panel’s workshop report

^d^CMS: Centers for Medicare & Medicaid Services

^e^PlumX Metrics: Research analytics tool that tracks online interactions with research articles (plumanalytics.com)

^f^PAMED: Commonwealth of Pennsylvania, Pennsylvania Medical Society

^g^VA: U.S. Department of Veterans Affairs

^h^DoD: U.S. Department of Defense

^i^Congress.gov: Official website for U.S. federal legislative information

^j^IPRCC: Interagency Pain Research Coordinating Committee

^k^ONDCP: Office of National Drug Control Policy
